# Mortality by opioid poisoning in children and teenagers and opioid prescriptions

**DOI:** 10.1186/s12887-021-03061-9

**Published:** 2021-12-13

**Authors:** Elise Cranfield, Elizabeth Ashcroft, Patrice Forget

**Affiliations:** 1 Edinburgh Medical School, College of Medicine and Veterinary Medicine, Edinburgh, EH16 4TJ UK; 2 University of Aberdeen, Aberdeen, AB25 2ZD UK; 3 Institute of Applied Health Sciences, Epidemiology group, School of Medicine, Medical Sciences and Nutrition, University of Aberdeen; Department of Anaesthesia, NHS Grampian, Aberdeen, AB25 2ZD UK

**Keywords:** Opioid, Poisoning, Children, Teenagers, Prescriptions

## Abstract

**Background:**

No comparisons between mortality from opioids in children and teenagers and opioid prescription patterns have been made in England.

**Aim:**

To investigate if an association exists between mortality rates from opioid poisoning in persons aged 19 years old and under and community opioid prescription in England.

**Methods:**

A retrospective analysis was undertaken for 2016 to 2019, comparing community opioid prescriptions and mortality rates from opioid poisoning.

**Results:**

The number of opioid prescriptions decreased over the study period (− 2.4%, *p* < 0.001). Most deaths due to opioid poisoning in children and teenagers were seen in those under one year old and those aged between 15 and 19 years old (Kruskal-Wallis: *p* = 0.12; Dunn’s test: *p* = 0.01). Deaths in all age ranges did not change significantly (Poisson Regression Analysis: *p* > 0.05).

**Conclusion:**

Despite the reduction in community opioid prescriptions, there was no decrease in the number of deaths in children and teenagers due to opioid poisoning.

**Supplementary Information:**

The online version contains supplementary material available at 10.1186/s12887-021-03061-9.

## What is known about this topic

No comparisons between mortality from opioids in children and teenagers and opioid prescription patterns have been made in England.

## What this study adds

This retrospective analysis shows that, despite the reduction in community opioid prescriptions, there was no decrease in the number of deaths in children and teenagers due to opioid poisoning.

## Introduction

Analgesics account for 20% of all cases of poisoning in children under 14 years old [1]. Similarly to adults, increasing mortality in children and young people from opioid poisoning parallels prescription patterns in numerous countries worldwide, such as Canada [2] and the United States [3]. No similar comparisons have been made for England to date, and this study aims to amend this information deficit by examining opioid poisoning in persons aged 19 or under by age group, type of opioid, and the correlation with the community opioid prescriptions in England.

To investigate if an association exists between the number of community prescriptions of opioids and the annual number of deaths by opioid poisoning in children and teenagers in England.

## Methods

### Study design

This is a retrospective analysis of prospective English databases between the years 2016 and 2019 comprising prescriptions of opioids and mortality related to opioid poisoning. Ethical approval and consent to participate were not applicable. All data was anonymised. There was no access to the autopsy reports to confirm the exact opioid considered the cause of death.

### Participants

This study includes those persons aged 19 and under in England (children and teenagers) who were recorded by the *Office for National Statistics (ONS)* as having died due to poisoning between 2016 and 2019.

### Data sources

The number of prescriptions of opioid analgesics between 2016 and 2019 was taken from *OpenPrescribing.net*. This database uses monthly files published by the NHS Business Service Authority, under the terms of the Open Government Licence as its source of prescribing data. Annual datasets between 2016 and 2019 for deaths registered in England from the *ONS* were used for mortality rates related to opioid poisoning over time and by age and the agent considered as the cause of death.

### Bias

Efforts were made to minimise bias, by considering the entire national population and the most comprehensive data sources. Deaths due to heroin were excluded to retain the focus on prescribed opioids.

### Study size

Between 2016 and 2019, there were a total of 93,833,243 opioid community prescriptions in NHS England and there were 76 deaths due to opioid poisoning in persons aged 19 and under.

### Statistical methods

Descriptive analyses comprised the population characteristics in terms of age and estimated size from 2016 to 2019. Opioid prescriptions have been summarised as absolute numbers per 10^6^ capita.

Data distributions over time have been analysed using a Kruskal-Wallis test and mortality rates have been analysed using Poisson Regression Analyses.

A simple linear regression has been used to investigate the potential association between the number of deaths and the number of prescriptions.

The data were analysed using SPSS for Windows, version 25.0 (IBM Corp. Released 2017, Armonk, NY) and a *p*-value < 0.05 has been considered significant.

## Results

The population of England, adult and paediatric, increased slightly over the study period (+ 1.8% and + 1.3%, respectively, between 2016 and 2019). The number of prescriptions showed a tendency to decrease over time (− 2.4% between 2016 and 2019, *p* < 0.001).

Data show a distribution of deaths due to opioid poisoning peaking in the middle-aged population (between 35 and 55 years old). This distribution did not change during the study period (Kruskal-Wallis: *p* = 0.39)(Supplementary Table S1, Supplementary Figs. S1 and S2).

Most deaths due to opioid poisoning in children and teenagers concerned children under the age of one and teenagers in the 15 to 19 year old group (Table 1). The only significant difference was for teenagers between 15 and 19 years old compared to the children aged between 5 and 9 years old (Kruskal-Wallis: *p* = 0.12; Dunn’s test = 0.01). As for adults, these distributions did not significantly change over the years (Poisson Regression Analysis: *p* > 0.05 for all the comparisons).

**Table 1:** Cause of death by age group in England between 2016 and 2019

Year	Age group	Cause of death			
		Methadone	Other opioids	Other synthetic narcotics	Other and unspecified narcotics
**2016**	**Under 1**	0	0	1	0
	**14**	1	0	0	0
	**59**	0	0	0	0
	**1014**	0	0	0	0
	**15–19**	2	6	2	1
	**Total**	**3**	**6**	**3**	**1**
**2017**	**Under 1**	0	0	0	0
	**14**	0	0	0	0
	**59**	0	0	0	0
	**1014**	0	1	0	0
	**15–19**	2	8	5	1
	**Total**	**2**	**9**	**5**	**1**
**2018**	**Under 1**	0	1	0	0
	**14**	2	0	1	0
	**59**	1	0	0	0
	**1014**	0	1	0	1
	**15–19**	0	3	2	1
	**Total**	**3**	**5**	**3**	**2**
**2019**	**Under 1**	0	1	0	0
	**14**	0	0	0	0
	**59**	0	0	0	0
	**1014**	0	1	1	0
	**15–19**	0	9	1	0
	**Total**	**0**	**11**	**2**	**0**

**Fig. 1:**
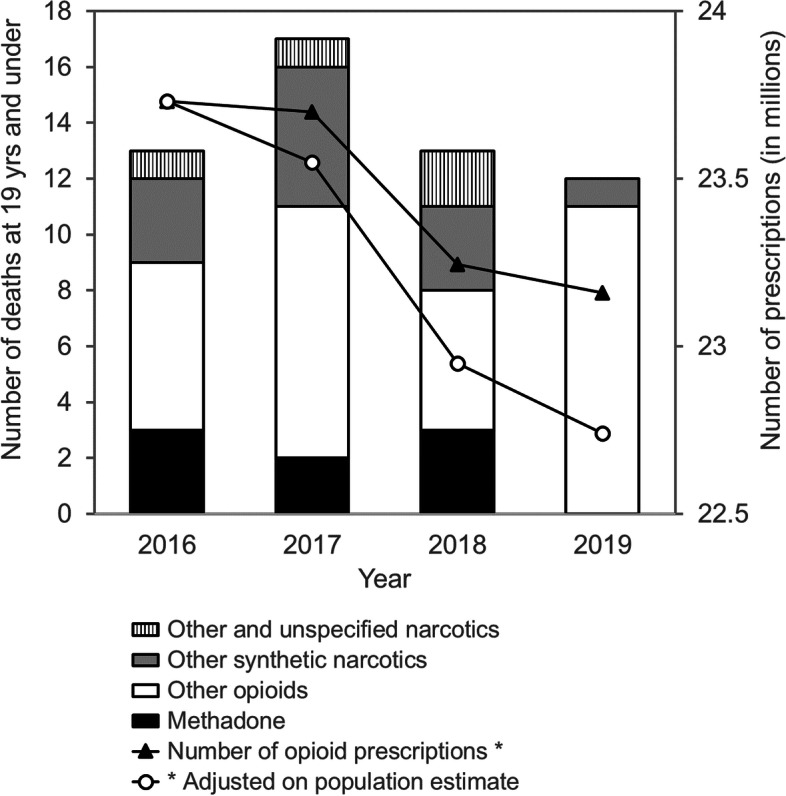
Number of deaths in children and teenagers in England compared to the number of community opioid prescriptions and the number of opioid prescriptions adjusted on the population estimates between 2016 and 2019

Number of deaths in children and teenagers, and type of molecules, were not related to the number of opioid prescriptions (adjusted on the population estimates) between 2016 and 2019 (Fig. 1 and Supplementary Figs. S3 and S4).

## Discussion

Most deaths due to opioid poisoning in children and teenagers were observed in those aged between 15 and 19 years old (Table 1). The seventeen deaths in the 15 to 19 year old group are the lift-off to the rising curve of deaths by opioids, and deaths in the 10 to 14 year old age group also increased over the study period. This may indicate that the upswing of deaths due to opioid poisoning starts in these younger age groups and justifies the need for early recognition, support, and dedicated services for teenagers. The 15 to 19-year-old age group includes young adults, given that a child is defined as a person under the age of 16 years by the Children Acts of Scotland and England. Young adults are a particularly vulnerable group meriting specific care as they transition across from paediatric to adult services. There is a decreasing rate of deaths due to opioid poisoning in children aged one to nine years old, which could be the result of various factors such as: like prescription patterns; support programmes for parents; child protection processes; and primary schools’ roles; and education about drug safety.

After adjustment per capita, another small peak was revealed involving children under one year old. Although there were only three deaths in the study period in this age group, this finding merits specific discussion. Infants develop fine motor skills during the first year of life: at five months old, an infant develops a palmar grasp, with a neat pincer grip developing by about at ten months old [4]. Gross motor milestones see the development of crawling and pulling to standing at nine months old and independent walking from nine to 18 months old [4]. These very limited developmental abilities of infants under the age of one year old raise questions as to how they reached, picked up, and ingested opioid preparations, particularly as these are dispensed in childproof containers. The recommendation arising from this study is that all deaths in under one-year olds warrant comprehensive investigation with emphasis on safeguarding issues.

Some caution must be expressed in the conclusions of this study as the age of the person receiving an opioid prescription is unknown. Additionally, without access to post-mortem results, it is impossible to confirm that a particular prescribed medication is the same as that involved in an individual poisoning. Other limitations are inherent within the data sources, including inaccurate recording or underreporting of deaths by opioid poisoning. Finally, an important limitation is the relatively low number of opioid poisoning in children and teenagers (at least in comparison to adults), challenging any multivariable modelling and also analysis of evolution over time. Moreover, four data points are a limited number to detect a change over time. The reasons for this choice are as follows: First, the data transmitted by the ONS have been aggregated on an annual basis only. Second, in an average year of study, if it were 14 deaths, it could be a significant number of months without deaths, indicating an excess of “zeros”, as well as “overdispersion” (variance greater than the mean), violating two assumptions of the Poisson regression, which is why we completed the analysis with a linear regression, to try to unmask the temporal trends. Although there are caveats to the conclusions reached by this study, one could argue that all these deaths in children and teenagers are preventable, and continued surveillance is necessary, in parallel with specific support services for these vulnerable age groups. To be more specific, future work could include a case-by-case study, to help understand how each death happened. This would be important for solving problems, which may or may not be similar for different age groups.

## Conclusion

Despite the decrease in community prescription, deaths due to opioid poisoning in children and teenagers remain problematic in England. No association has been confirmed between these two phenomena, which suggests that alternative strategies and specific support services are required for these vulnerable groups, monitored by continued data collection and evaluation.

## Supplementary Information

**Additional file 1.**

## Data Availability

The datasets generated and/or analysed during the current study are available in the files published by the NHS Business Service Authority: https://openprescribing.net/analyse/#org=regional_team&numIds=4.7.2&denom=nothing&selectedTab=chart and the Office for National Statistics: https://www.ons.gov.uk/peoplepopulationandcommunity/birthsdeathsandmarriages/deaths/datasets/deathsregisteredinenglandandwalesseriesdrreferencetables
