# Supplementary material for: Mortality by opioid poisoning in children and teenagers and opioid prescriptions
Source: BMC Pediatr. 2021 Dec 13;21:569. doi: 10.1186/s12887-021-03061-9 (PMC8667418; doi:10.1186/s12887-021-03061-9)
Supplement: Supplementary file 1 — Additional file 1. [file 12887_2021_3061_MOESM1_ESM.docx]

# Appendix

**Table S1:** Total number of opioid prescriptions, total number of deaths due to opioid poisoning, and mid-year population estimates in England between 2016 and 2019.


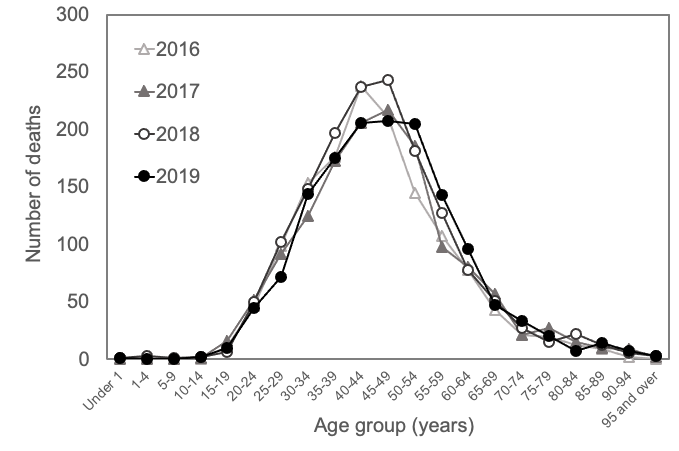
**Figure S1:** Distribution of deaths due to opioid poisoning by age group in England between 2016 and 2019.


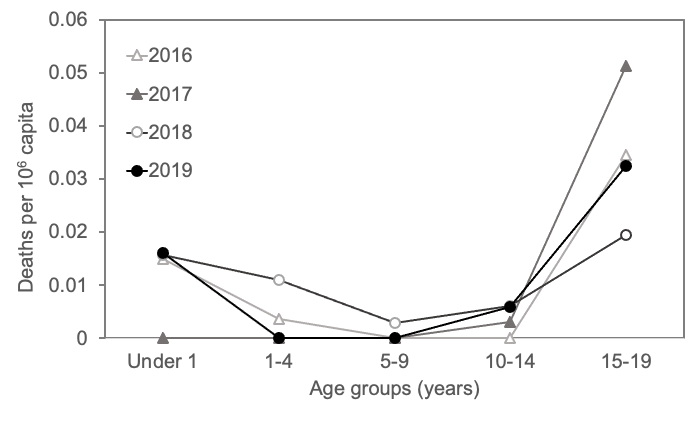


**Figure S2:** Distribution of deaths due to opioid poisoning by age group in children and teenagers in England between 2016 and 2019.

**Figure S3:** Number of community opioid prescriptions compared to the number of deaths in children and teenagers due to opioid poisoning in England between 2016 and 2019.

**Figure S4:** Number of community opioid prescriptions compared to the total number of deaths due to opioid poisoning in England between 2016 and 2019.
